# Coronary Perforation of Distal Diagonal Branch Followed by Prolonged Recurrent Cardiac Tamponade Finally Resolved with Pericardiotomy - the Potential Risk of Hydrophilic Guide-Wires

**DOI:** 10.2174/1874192401711010061

**Published:** 2017-06-19

**Authors:** Rafał Januszek, Krzysztof Bartuś, Radosław Litwinowicz, Artur Dziewierz, Łukasz Rzeszutko

**Affiliations:** 1 2^nd^ Department of Cardiology and Cardiovascular Interventions, University Hospital, Krakow, Poland; 2 Department of Cardiovascular Surgery and Transplantation, John Paul II Hospital, Krakow, Poland; 3 2^nd^ Department of Cardiology, Jagiellonian University Medical College, Krakow, Poland; 4 Department of Interventional Cardiology, Jagiellonian University Medical College, Krakow, Poland

**Keywords:** Coronary artery perforation, Hydrophilic guide-wires, Cardiac tamponade, Pericardiotomy

## Abstract

**Purpose:**

Coronary artery perforation (CAP) is a complication of percutaneous coronary interventions (PCIs). Hydrophilic guide-wires have been shown to increase the probability of CAP. Depending on the size of perforations we adopt different treatments.

**Case:**

We present the case of a 73-year old male with coronary artery disease and severe aortic valve stenosis. The patient was in the process of qualifying for a transcatheter aortic valve implantation. Unfortunately, CAP of the first diagonal branch of the LAD occurred during PCI. Initially, abrupt bleeding to the pericardial sac was primarily restrained. However, in the following days, pericardial bleeding became silent, prolonged and finally resulted in surgical pericardiotomy and surgical aortic valve replacement.

**Conclusion:**

This case depicts that in some cases, more aggressive endovascular treatment of CAP during the acute phase could decrease the probability of future radical surgical treatment. Although, in other cases, avoiding radical endovascular treatment of CAP and secondary necrosis along the distribution of the artery culminates in a higher risk for conversion to a surgical cardiac procedure. Accurate primary assessment of CAP seriousness and careful observation after PCI could improve results and lead to avoiding severe complications.

## 1 INTRODUCTION

The incidence of coronary artery perforation (CAP) during percutaneous coronary interventions (PCI) varies between 0.2-0.6%, and may rise to 3% when devices such as an atherotome or laser are used [1]. It is estimated that in about 20% of cases, the angioplasty guide-wire is the cause of CAP. According to Ellis *et al.*, CAP can be classified into one of three types: type I: extraluminal crater without extravasation; type II: pericardial or myocardial blushing without contrast streaming; type III: extravasation through perforation >1 mm with contrast streaming, and subtype III - cavity spilling (CS), when the extravasation streams toward an anatomic chamber such as the coronary sinus, the atria orventricles.[2]. CAP may be contained and may also cause serious acute complications such as cardiac tamponade, myocardial infarction, malignant arrhythmias and death [2]. Cardiac tamponade occurs in 11-46% of patients experiencing CAP after PCI [3]. It increases in higher Ellis stages of CAP. About 30% of these patients require emergency surgery [3]. Hydrophilic polymer-coated guide-wires are very useful for crossing areas of severe stenosis but are associated with a higher risk of CAP, especially when trying to cross complex lesions such as: occlusions, bifurcations, long lesions and tortuous vessels [1, 4, 5]. Depending on the class of CAP and bleeding intensity it could be treated less or more invasively. In mild cases, protamine infusion or catheter balloon treatment is used [1, 4]. Other possible treatments include: covered stents, coils and organic substances for artery occlusion [1, 4].

## 2 CASE

**Fig. (1):**
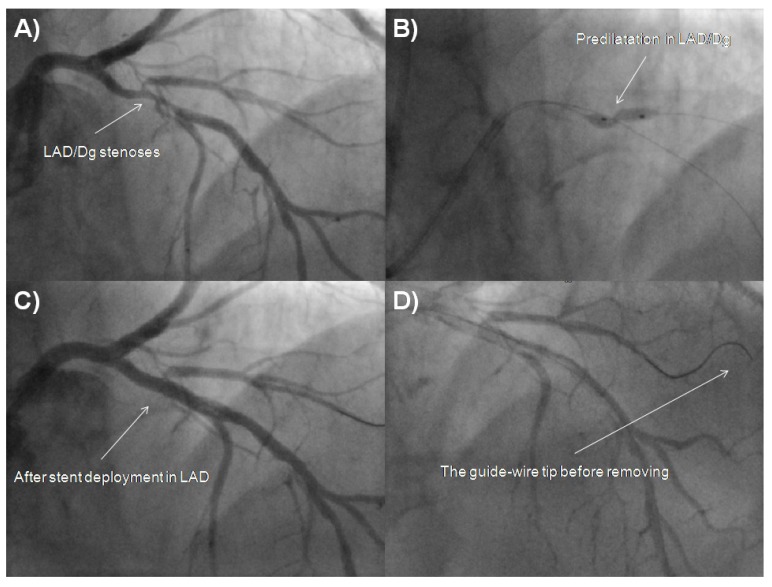
Fluoroscopic images of left coronary artery during PCI. **A** - Cine image of LAD/Dg stenosis. **B** - Cine image of LAD/Dg stenoses during predilatation. **C** - Cine angiography of LAD/Dg after PCI. **D** - The guide-wire tip before removal from Dg. Dg, diagonal branch; LAD, left anterior descending; PCI, percutaneous coronary intervention.

**Fig. (2):**
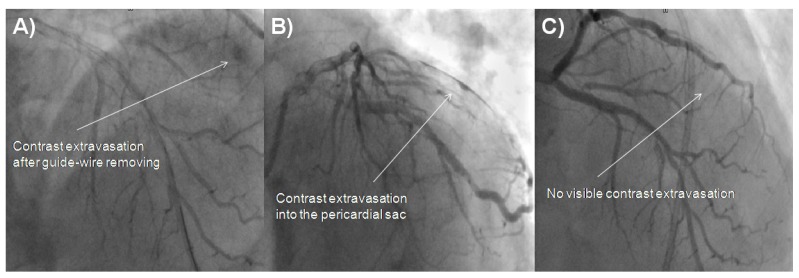
Fluoroscopic images of Dg perforation. **A** - Contrast extravasation projecting distal part of Dg. **B** - Contrast extravasation in the pericardial sac. **C** - The cessation of contrast extravasation in cine angiography. Dg, diagonal branch.

A 73-year-old male was admitted due to severe symptomatic aortic stenosis for further diagnosis and treatment. The patient demonstrated with hypertension and history of ischaemic cerebral stroke 2 months prior to presentation. An echocardiography confirmed severe aortic stenosis with an aortic valve area of 0.4-0.5 cm^2^ and the transvalvular gradient of 74/48 mmHg. The left ventricle ejection fraction was 60%, with mild hypokinesis of the anterior wall and anterior part of the interventricular septum. Carotid artery ultrasound revealed critical stenosis of the left internal carotid artery (95%). Coronary angiography showed 80% stenosis of the left anterior descending artery (LAD) in the proximal part including the first diagonal branch (Dg) (Fig. **1A**). After coronary angiography, the case was discussed among our “Heart team” and the patient was scheduled for subsequent transcatheter aortic valve implantation, percutaneous coronary intervention (PCI) of the proximal LAD and carotid artery stenting. PCI of the LAD was performed. One 6 French Extra Backup 4.0 guide catheter was used, a BMW Universal II (Abbott Vascular, Santa Clara, California, USA) guide-wire was placed in the LAD, and after several attempts with other non-hydrophilic wires, ASAHI Sion Black (Abbott Vascular, Santa Clara, California, USA) was placed in the first diagonal branch. Predilatation with a balloon catheter (3.0 x 12 mm 10 atm.) was performed in the proximal Dg (Fig. **1B**). Afterwards, we implanted a DES Resolute stent (3.5 x 22 mm in LAD 16 atm.) (Fig. **1C**). The Proximal Optimization Technique (POT) was performed with a non-compliant balloon catheter (3.5 x 6 mm up to 20 atm.) We visualized a type B dissection in the proximal edge of the stent which we placed in the LAD. There was no contrast extravasation before removal of the guide-wire located in the Dg (Fig. **1D**). Immediately after removing the guide-wire from the Dg, contrast extravasation appeared distally (Figs. **2A**, **2B**). Due to the increase of clinical features of cardiac tamponade (drop in blood pressure to 80/60 mmHg) and in subsequent coronary angiographies, there was no response to pharmacological treatment, pericardiocentesis was performed. A total of 300 ml of blood was removed with resolution of clinical tamponade symptoms. After clinical stabilization, a Fielder XT-A guide-wire was put into the Dg. Afterwards, two unsuccessful passages through stenotic lesions were attempted with 1.5 x 15 mm and 1.25 x 10 mm balloon catheters. The same difficulties appeared with the use of a Finecross micro-catheter. Due to the hemodynamic destabilization, we decided to finish the procedure and give the patient 30 mg of protamine sulphate. Over the next several hours after the procedure, 180 ml of blood was removed from the pericardium. Two days after the procedure, pericardial effusion stabilized and the pericardial catheter was removed. Due to progressive anaemia, the patient was transfused 2 units of packed red blood cells 8 days after the CAP. Increased pericardial blood was noted 10 days after the procedure and the pericardial sac was punctured again resulting in the removal of 680 ml of blood. In the following three days, 440 ml and 480 ml were removed from the pericardium. Fifteen days after the PCI coronary, the angiography revealed no signs of contrast extravasation (Fig. **2C**). The patient was discussed by the “Heart team” and scheduled for surgical revision of the pericardial sac, concomitant aortic valve replacement surgery and left internal carotid artery endarterectomy, scheduled for the sixteenth day after the PCI. During surgical revision, several clots, haemolyzed blood and signs of pericardial adhesions and inflammation were seen in the pericardium. The operation was performed with hypothermia (32^°^C), extracorporeal circulation and crystalline cardioplegia. After the surgical opening of the ascending aorta, the pathologic aortic valve was removed and replaced with a new biological aortic valve Medtronic Hancock II 23 A (Medtronic Minneapolis, MN, USA). Decompression of the tamponade was performed and clots were removed. Interestingly, bleeding from the entire surface of the pericardium and visible signs of inflammation were seen. Laboratory tests did not show decreased platelets (165,000/µl), prothrombin time was 14.1 sec, and activated partial thromboplastin time was 40.5 sec on the day of surgery. During the cardiac surgery and postoperative period, the patient required transfusion of 6 units of packed red blood cells. The postoperative period was uneventful. The patient was discharged 7 days after the operation and transferred back to the referring hospital for further treatment.

## 3 DISCUSSION

Stathopoulos *et al.* demonstrated that among 23,399 patients after PCI, CAP occurred in 73 patients (0.31%) [4]. CAP rates were recorded for guide-wire related CAP in 31 patients, balloon/stent-related in 46.5%, cutting the balloon in 5.47%, rotational atherectomy devices in 4.1% and other causes in 1.37% of patients [4]. Among the culprit guide-wires which caused CAP, 38.7% were “workhorse” guide-wires, 22.6% were hydrophilic guide-wires and 38.7% were stiff guide-wires. Other researchers have reported that a hydrophilic wire was considered the culprit in the majority of guide-wire induced CAP cases with the frequency ranging from 50 to 100% [6]. However, some studies demonstrated that non-hydrophilic guide-wires were mostly responsible for CAP [3]. Teis *et al.* demonstrated that the use of hydrophilic guide-wires was an independent risk factor of CAP for the treatment of chronic total occlusions (CTO) [5]. Hydrophilic guide-wires cause increased the risk of CAP due to their low coefficient of friction and ease of distal migration [7].

Nowadays, in difficult and lengthy procedures, we strive to reduce radiation doses. Modern x-ray cameras have the ability to reduce the radiation dose without restricting the visual field. In older devices it is only possible to reduce the collimation beam and the field of view. Managing radiation exposure with older devices could result in poor visibility of the peripheral parts of a guide-wire during the procedure. The tendency for hydrophilic guide-wires to migrate is dangerous, and due to this fact, it is necessary to control the position of its distal parts during the procedure. Teis *et al.* observed increased rates of delayed passage of blood after CAP with hydrophilic guide-wires which is usually due to small perforation [5]. The mortality and morbidity rate after CAP reaches up to 7% [8]. During medium and long-term follow-ups, the major adverse cardiovascular events (MACE) incidence was up to 41% and mortality rate could rise to 17% [3]. After PCI of class III CAP, mortality rises even to 19% [1]. Conventional management to treat CAP includes prolonged balloon inflation and anticoagulation reversal with protamine [7]. Protamine use in patients with CAP seems to be safe without increased rates of vessel/stent thrombosis [9]. Prolonged balloon inflation with a perfusion balloon catheter is the ideal solution for managing CAP. Type I and II perforations are predominately caused by stiff or hydrophilic guide-wires and tend to heal spontaneously, often requiring no more than observation [1, 2, 5, 6]. However, some cases of type II CAP have the potential to progress into tamponades [2]. In type I and II perforations, if there are significant extravasations, prolonged balloon inflation is beneficial [2]. If prolonged balloon inflation fails or there is a large type III perforation, covered stents have to be deployed immediately to seal the perforation and prevent haemopericardium [10]. In mild cases, deployment of a standard stent with narrow struts seems to be sufficient to seal the CAP [11]. In our case of type II CAP, we were unable to deliver a balloon catheter. During the procedure we had to implant a pericardial catheter. Due to patient stabilization and sustained muscle heart contractility in the Dg supplied area, we decided not to implant a covered stent into the LAD. As we had difficulties with balloon catheter delivery, we concluded that delivery of a covered stent would also be unsuccessful due to bulkiness and lack of flexibility of the stent. Also, deployment of a covered stent in LAD would induce relatively large muscle necrosis. Covered stents serve as optimal treatment for CAP occurring in large epicardial arteries involving proximal and mid segments [10]. Recently, highly deliverable pericardial covered stents have been used in CAP [12]. If covered stents fail, patients require urgent surgical intervention, which is accompanied by high morbidity and mortality [5]. The incidence of subacute thrombosis and restenosis associated with covered stents is relatively higher than in the case of standard stents [13]. Another option is new generation pericardial-covered stent implantation [12]. Operative repair of CAP includes either ligation or suturing of the vessel for haemostasis and bypass grafting to the distal vessel [14]. In addition, pericardial patch/teflon felt wrapping repair of the perforation with or without coronary artery bypass grafting (CABG) is advocated especially when multiple stents with CAP and subepicardial haematoma are present [14]. The use of metal coils, gel foam or other embolization materials is another option for endovascular treatment of CAP [14]. It was confirmed that pericardial drain insertion, usually for class III CAP, was a strong risk factor for subsequent death [1]. However, in patients with class II CAP, there were no deaths and no need for pericardial drain insertion or urgent CABG [1]. The seriousness of class III CAPs was confirmed in several studies, and in most cases of class III CAP (all pericardiocentesis) – patients underwent emergency bypass surgery and died [3].

## CONCLUSION

This case suggests that in some cases, more aggressive endovascular treatment of CAP during the acute phase could decrease the probability of future radical surgical treatment. However, conservative options for the percutaneous treatment of CAP, the aim of which is to avoid necrosis of the heart muscle, is associated with increased risk of a subsequent need for surgical intervention in the future. Accurate primary assessment of CAP seriousness and careful observation after PCI could improve results and lead to avoiding severe complications.
